# Extracranial propagation of glioblastoma with extension to pterygomaxillar fossa

**DOI:** 10.1186/1477-7819-9-53

**Published:** 2011-05-19

**Authors:** Damir Tomac, Darko Chudy, Smiljka Lambaša, Iva Topić, Gordan Grahovac, Arijana Zoric

**Affiliations:** 1 Department of Neurosurgery, Clinical Hospital Dubrava, Zagreb, Croatia; 2 Department of Pathology, Clinical Hospital Dubrava, Zagreb, Croatia; 3 Department of Otorhinolaryngology, Head and Neck Surgery, Clinical Hospital Center Zagreb, Croatia; 4 Rudjer Boskovic Institute, Division for Molecular Medicine, Laboratory of Molecular Oncology, Zagreb, Croatia

## Abstract

**Background:**

Glioblastoma multiforme is a highly malignant primary brain tumor that shows marked local aggressiveness, but extracranial spread is not a common occurrence. We present an unusual case of recurrent glioblastoma in 54-year old male that spread through the scull base to the ethmoid and sphenoid sinuses, to the orbita, pterygomaxillar fossa, and to the neck.

**Methods:**

A 54-year old male underwent left temporal resection because of brain tumor of his left temporal lobe. Operation was followed by external beam radiation combined with temozolomide. The tumor recurred eight months after first surgery. The patient developed swelling of left temporal region, difficult swallowing and headache. MRI of head showed recurrent tumor, which invaded orbita, ethmoid and sphenoid sinuses, nasal cavity, pterygomaxillar fossa.

**Results:**

The patient died ten months after initial diagnosis of glioblastoma multiforme, and two months after his second operation.

**Conclusions:**

The aggressive surgical operation helped to downsize the tumor mass as much as possible, but did not prolonged significantly the life or improved the life quality of the patient. The current literature is reviewed, and the diagnostic approaches as well as therapeutic options are discussed.

## Background

Glioblastoma multiforme is a highly malignant primary brain tumor. The median survival with therapy is approximately 9-12 months [1]. Glioblastoma shows marked local aggressiveness, but extracranial spread is not a common occurrence. It is believed that dura provides excellent protection against infiltration by malignant tumors. Improvement of treatment options and survival time led to increase of extracranial recurrence of glioblastoma. Most commonly glioblastomas metastases are to the lungs, lymph nodes, liver, and bones [2].

We report an exceptional case of glioblastoma multiforme spreading extracranially to the orbita, ethmoid and sphenoid sinuses, nasal cavity, pterygomaxillar fossa, and neck.

## Case Report

**Figure 1:**
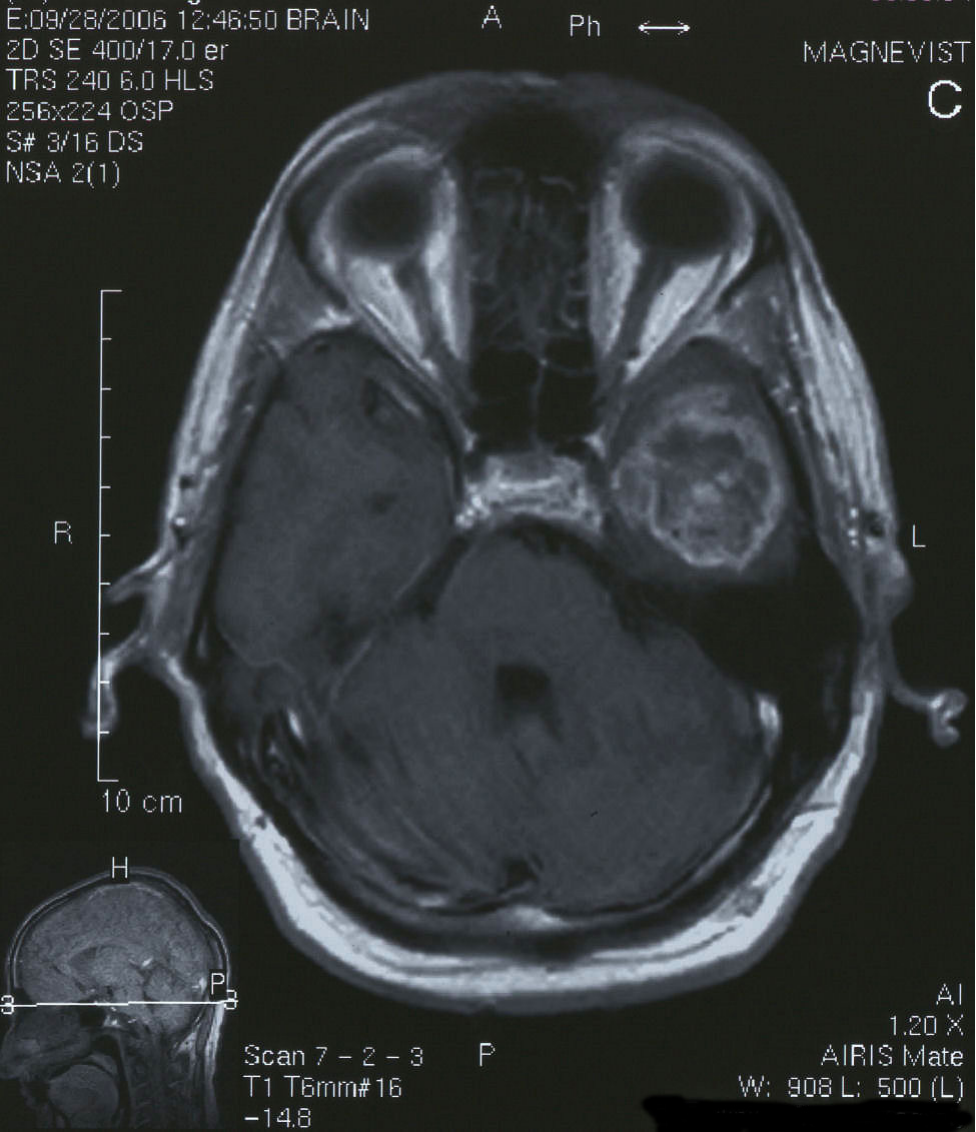
Axial T1 weighted contrast enhanced MRI image demonstrating ring-enhanced lesion of the left temporal lobe

**Figure 2:**
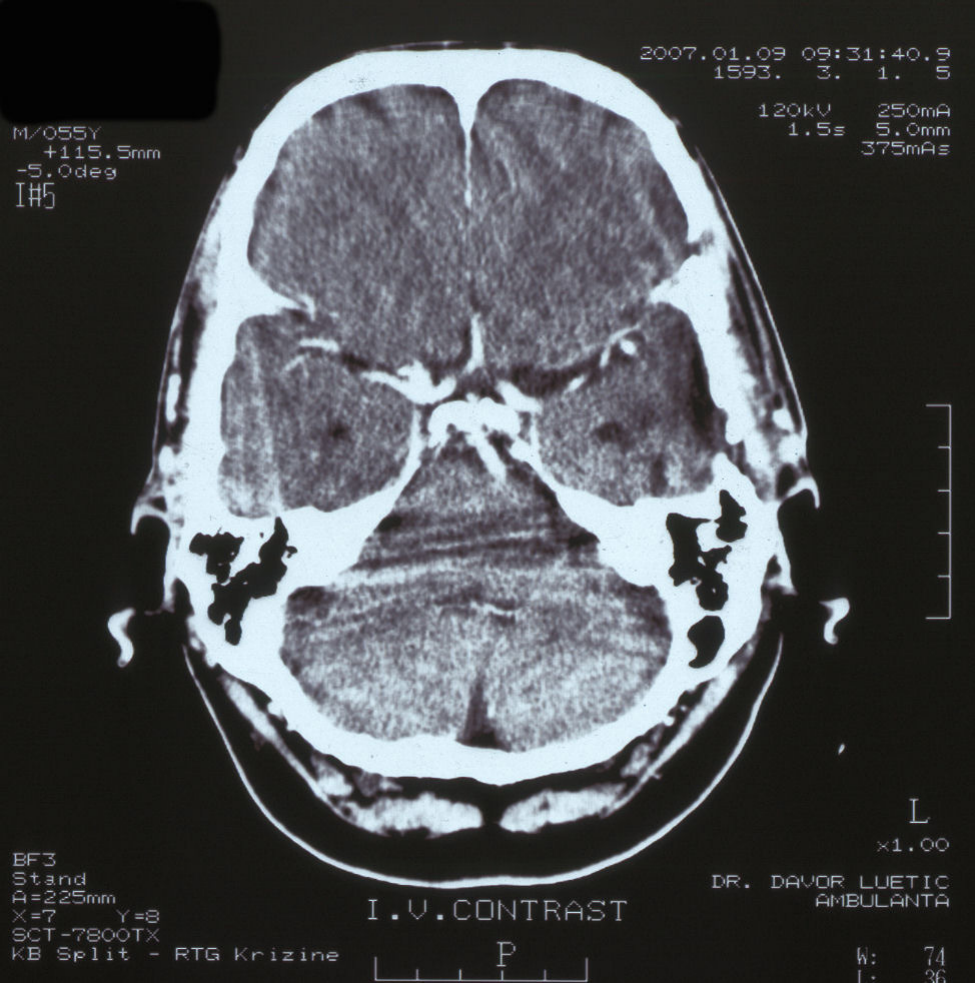
**Axial contrast enhanced MSCT of the head showing no signs of tumor**.

A 54-year old Caucasian male presented to Department of Neurosurgery complaining of severe headaches, dizziness, and dysarthria that lasted for 2 weeks. His personal and family history was unremarkable, and his Karnofsky score was 90. Upon admission MSCT showed a hypodense lesion in the left temporal lobe. After initial analysis MRI of the head was scheduled, which showed a ring-enhancing lesion in the left temporal lobe.

(Figure 1) We preformed left temporal osteoplastic craniotomy, and tumor was removed along with the surrounding normal brain tissue (Figure 2). Regression of dysarthria was noticed after the operation, and his Karnofsky score was 100 at discharge. Histopathological analysis confirmed diagnosis of glioblastoma, gradus IV according to WHO. After discharge patient was sent for oncological evaluation.

The patient received radiotherapy in daily factions of 2 Gy given 5 days per week for 6 weeks, for total 60 Gy plus continuous daily temozolomide (75 mg per square meter of body-surface area per day). Following with the six cycles of the adjuvant temozolomide therapy (150 to 200 mg per square meter for 5 days).

**Figure 3:**
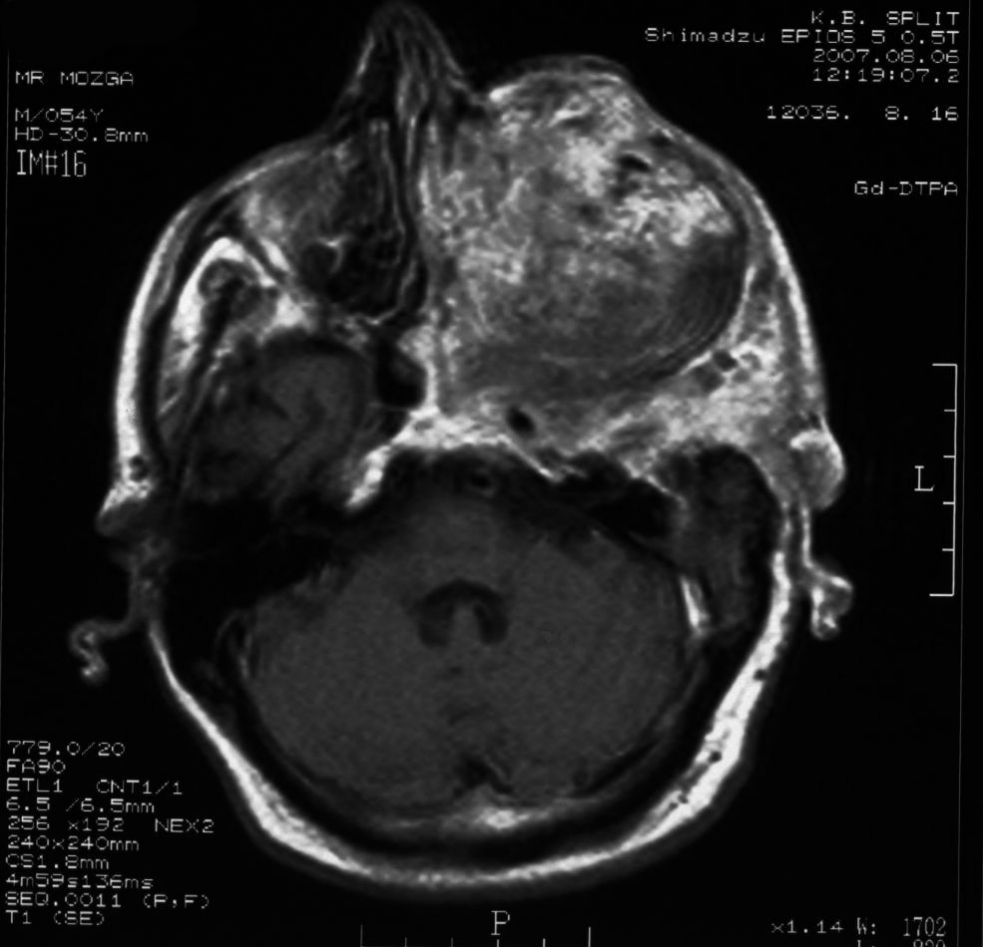
**Axial T1-weighted contrast enhanced MRI image demonstrates extra cranial portion of the tumor extended to the sphenoid and ethmoid sinuses, nasal cavity, and orbit**.

**Figure 4:**
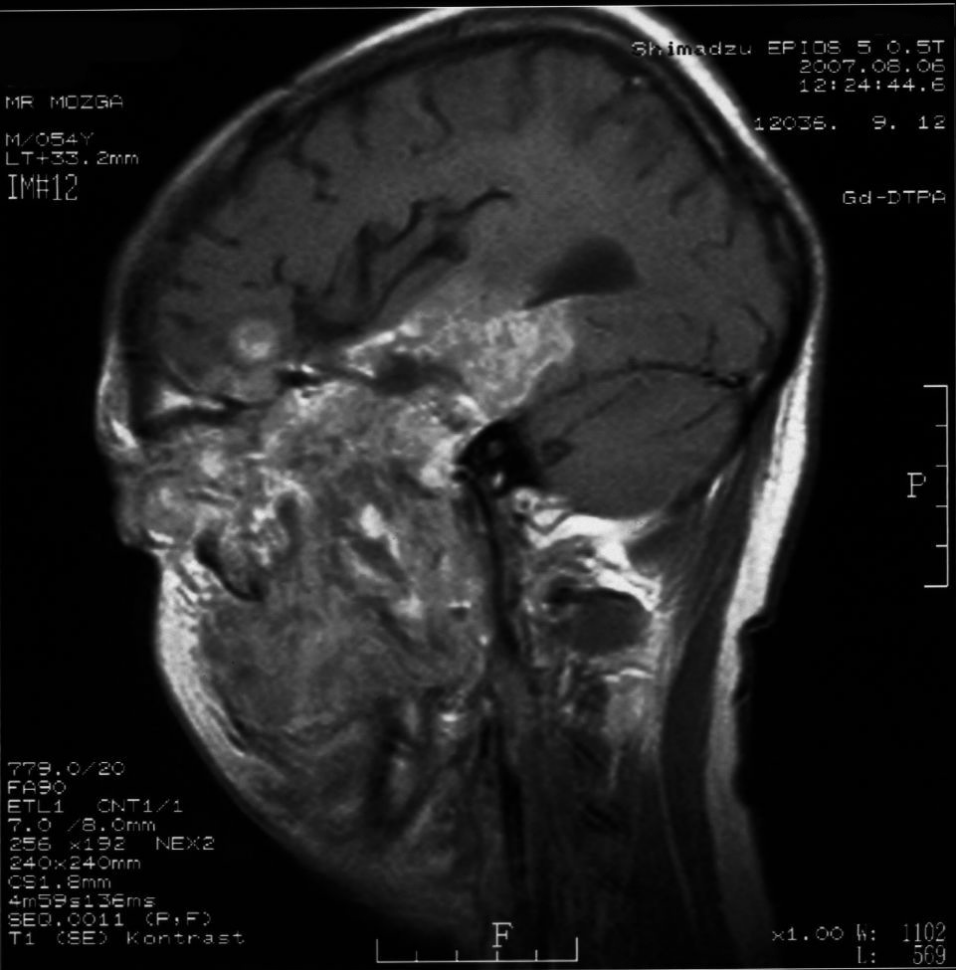
**Sagittal T1-weighted contrast enhanced MRI image demonstrates intracranial and extra cranial portion of the tumor extended to the sphenoid and ethmoid sinuses, nasal cavity, orbit, pterygomaxillar fossa, and neck**.

**Figure 5:**
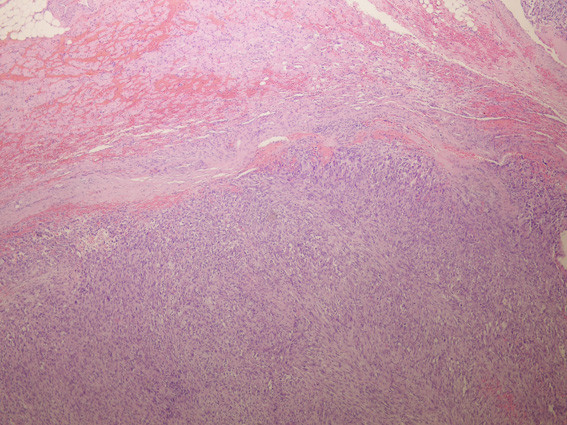
**Tissue sections showing a glioblastoma multiforme with invasion in muscle tissue (hematoxylin-eosin stain, ×400)**.

**Figure 6:**
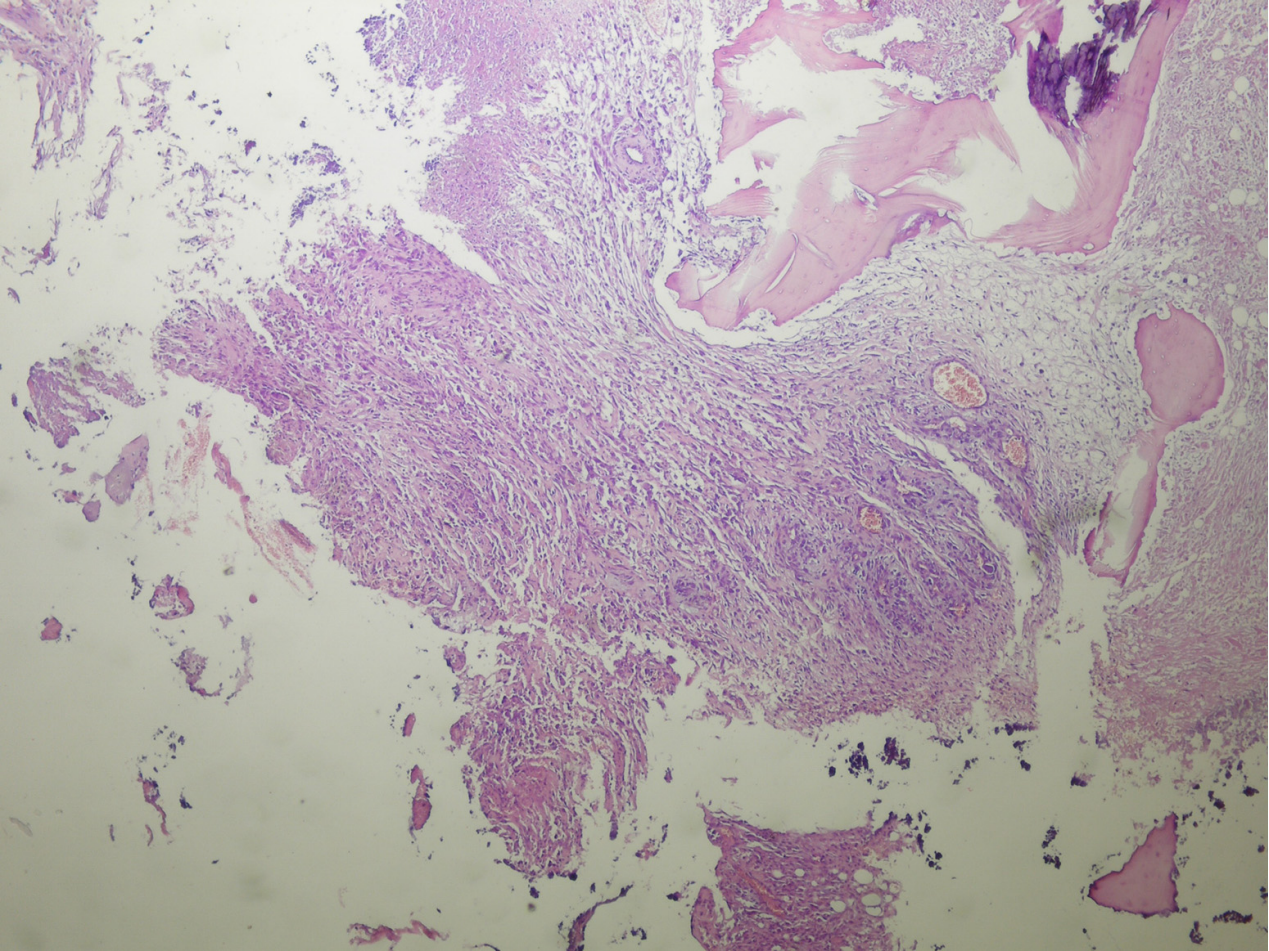
Tissue sections showing a glioblastoma multiforme with bone invasion (hematoxylin-eosin stain, ×400)

**Figure 7:**
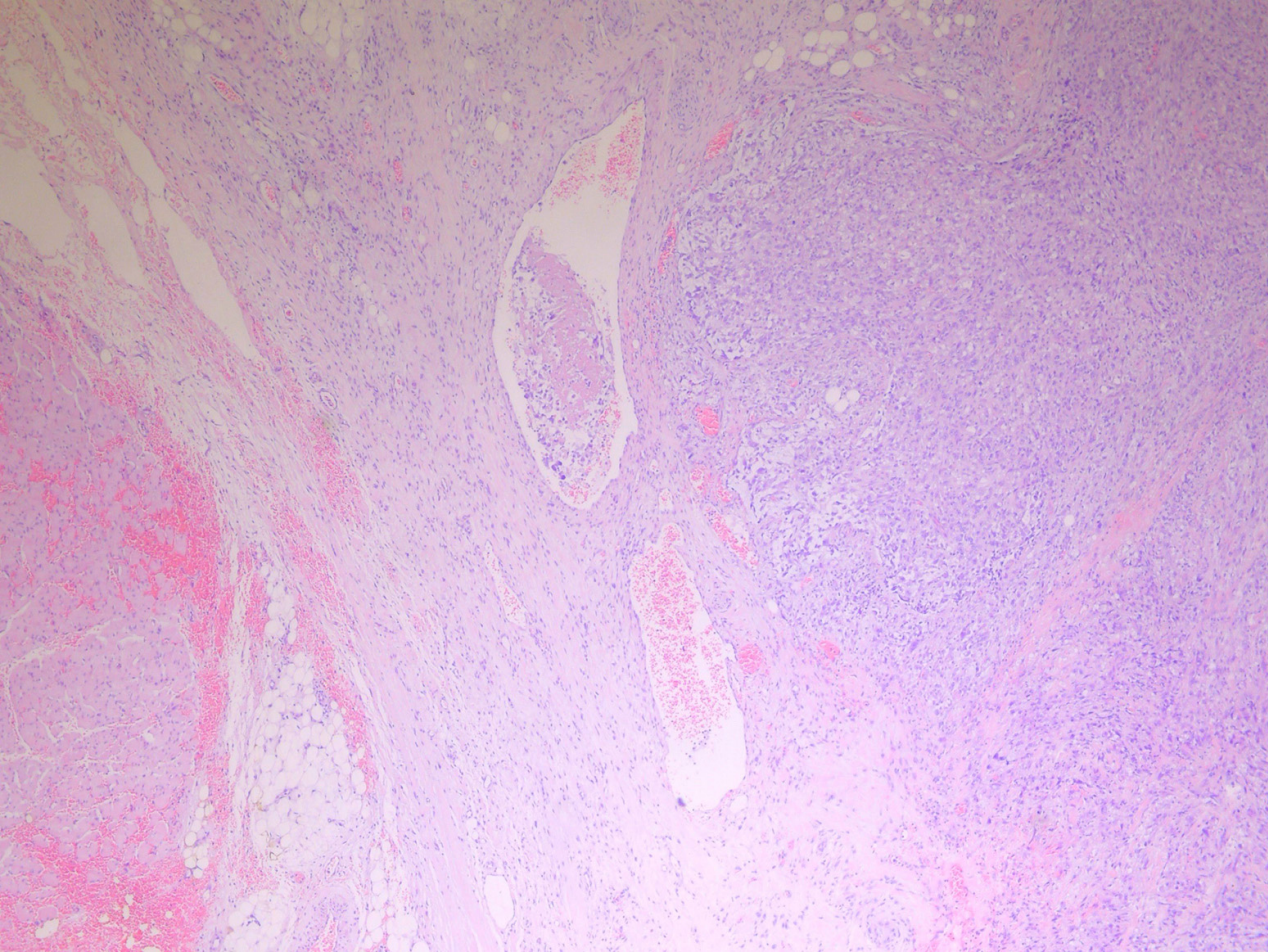
**Tissue sections showing a glioblastoma multiforme with angioinvasion (hematoxylin-eosin stain, ×400)**.

Three months after operation control MSCT showed no signs of tumor. Eight months after the first operation MRI was performed and revealed a tumor that involved middle cranial fossa with extension to the left orbita, ethmoid and sphenoid sinuses, nasal cavity, and pterygomaxillar fossa (Figure 3, Figure 4). The patient had Karnofsky score of 70 at second admission when he was transferred to Department of Neurosurgery. Left temporal recraniotomy and reduction of intracranial tumor and tumor in pterygomaxillar fossa was performed. Zygoma and left side of the mandible were resected. Parotid gland and masseter muscle were used for defect reconstruction. Histological analysis showed glioblastoma multiforme with invasion of bone, muscles, and blood vessels (Figure 5, Figure 6 and Figure 7). The patient died two months after second operation. The patient and the family declined any other oncology treatment because the Karnofsky score at discharge was 40, which rapidly deteriorated after discharge form the hospital. Autopsy was not performed.

Despite malignant nature of glioblastoma multiforme, extracranial metastases are rare [3]. Glioblastoma are prevented from metastasing by the relatively impassable dura, tough basal membrane around intracerebral blood vessels, and lack of true lymphatics in the brain[4]. Local dissemination to the scalp, face, and neck usually occurs after operations and failure of closure of the dura, or after shunt operations. Such procedures can facilitate tumor cells to enter vascular system, extracranial lymphatic system, or directly enter the peritoneum in the setting of a ventriculoperitoneal shunt. Direct bone invasion, which might interfere with local dural blood supply resulting in dural necrosis, is also possible. According to pathohistological findings in our patient, we believe that extracranial spread was caused by angioinvasion and invasion of the bone of the scull base. Similarly, surgery may also have made metastases more likely by simply prolonging the life of the patient [5]. Although rare, there are cases of glioblastoma multiforme with extracranial metastases in the absence of previous craniotomies [2,6]. Various mechanisms of spontaneous transdural spread have been described. The tumor can extend through the perivascular or dural slit, the increase of the intracranial pressure over a long period of time will allow the cerebral cortex to insinuate itself wherever possible through the dura, or transdural extension may originate by infiltration of tumor cells into the previously herniated normal brain substance [7]. The tumor can also pass through the dura mater by way of cranial or spinal nerves [8], or the dura can be directly destroyed by the tumor.

## Conclusions

In summary, the extra cranial spread of glioblastoma multiforme is a rare occurrence. In this report we presented unusual case of extra cranial spread of glioblastoma multiforme after resection and concomitant radiotherapy with chemotherapy. The tumor showed aggressive clinical behavior after standard treatment in very short period of time. The recurrent tumor extended to the orbit, ethmoid and sphenoid sinuses, and to the pterygomaxillar fossa. During second surgery we could observe the adherent brain tumor mass to the temporal dura. The tumor destructed the floor of the skull base; the dura mater was absent from the floor of the skull base. The pathological findings revealed invasion of the bone of the skull base, and underlying masticator muscles in the infratemporal fossa. Due to intraoperative and histological findings we believe that the tumor spread with direct invasion of the dura and underlying scull base and mastication muscles. In the later stage of disease tumor showed angioinvasion. Aggressive operation can downsize the tumor mass but the life quality may not be improved significantly.

## Consent

Written informed consent was obtained from the patient for publication of this Case report and any accompanying images. A copy of the written consent is available for review by the Editor-in-Chief of this journal.

## List of abbreviations

MRI: magnetic resonance imaging; MSCT: multi slice computed tomography; WHO: World health organization

## Competing interests

All authors declare that they do not have any financial or non-financial competing interests in relation in relation to this manuscript.

## Authors' contributions

DT collected the data, analyzed data and wrote the paper, DC gave conceptual design and edited the paper, SL gathered pathological pictures and interpreted them, IT supervised and edited the paper, GG wrote the paper, AZ supervised the paper end edited the paper. All authors read and approved the final manuscript.
